# *Rpd3* interacts with insulin signaling in *Drosophila* longevity extension

**DOI:** 10.18632/aging.101110

**Published:** 2016-11-14

**Authors:** Jared K. Woods, Tahereh Ziafazeli, Blanka Rogina

**Affiliations:** ^1^ Department of Genetics & Genome Sciences, School of Medicine, University of Connecticut Health, Farmington, CT 06030, USA; ^2^ Institute for Systems Genomics, School of Medicine, University of Connecticut Health, Farmington, CT 06030, USA; ^3^ current address: Division of Pediatric Endocrinology, Department of Pediatrics, Faculty of Health Sciences, McMaster University, Ontario, Canada

**Keywords:** Rpd3, Insulin/Insulin-like signaling, aging, Drosophila melanogaster

## Abstract

Histone deacetylase (HDAC) 1 regulates chromatin compaction and gene expression by removing acetyl groups from lysine residues within histones. HDAC1 affects a variety of processes including proliferation, development, metabolism, and cancer. Reduction or inhibition of Rpd3, yeast and flyHDAC1 orthologue, extends longevity. However, the mechanism of *rpd3*'s effects on longevity remains unclear. Here we report an overlap between *rpd3* and the Insulin/Insulin-like growth factor signaling (IIS) longevity pathways. We demonstrated that *rpd3* reduction downregulates expression of members of the IIS pathway, which is associated with altered metabolism, increased energy storage, and higher resistance to starvation and oxidative stress. Genetic studies support the role of IIS in *rpd3* longevity pathway, as illustrated with reduced stress resistance and longevity of flies double mutant for *rpd3* and *dfoxo,* a downstream target of IIS pathway compared to *rpd3* single mutant flies. Our data suggest that increased *dfoxo* is a mediator of *rpd3*'s effects on fly longevity and intermediary metabolism, and confer a new link between *rpd3* and IIS longevity pathways.

## INTRODUCTION

Histone deacetylase (HDAC) proteins are highly conserved enzymes whose major role is regulation of chromatin structure. Histone deacetylation increases chromatin compaction with subsequent inhibition of gene transcription. Class I HDACs are zinc-dependent histone deacetylases and include HDACs 1, 2, 3, and 8. HDAC1 affects a variety of processes such as proliferation, differentiation, and development [1, 2]. Disruption of HDAC1 function has been associated with many disorders including cancer and neuro-degenerative diseases including Alzheimer's disease [3, 4]. HDAC inhibitors are now used clinically for the treatment of several disorders including malignancies [5]. Rpd3, an orthologue of mammalian HDAC1, can also deacetylate lysine residues of various other proteins, such as P53, or bind to promoter region of other genes (*p21* and *p57*) and affect their transcription [6]. Reduced expression of Rpd3 extends lifespan in yeast, worms and fruit flies [7–12]. Similar effects on *Drosophila* lifespan were achieved by feeding fruit flies 4-phenylbutyrate (PBA), a histone deacetylase inhibitor [3]. Despite all of these studies, the mechanism of the beneficial effects of decreased *rpd3* on longevity remains poorly understood.

The Insulin/insulin-like growth factor signaling (IIS) pathway is a nutrient-sensing pathway that regulates growth and development, energy homeostasis, stress response, and reproduction. Notably, mutations that reduce IIS activity are associated with longer lifespan in yeast, worms, flies, and mice [14–17]. *Drosophila* has eight insulin-like peptides (Dilps) that activate downstream events by binding to the insulin receptor [18]. dFOXO is the downstream target of IIS in flies [17]. When IIS is active, dFOXO is phosphorylated by dAkt, which leads to its binding to 14-3-3 proteins and its degradation. Reduced IIS results in decreased phosphorylation of dFOXO that promotes dFOXO nuclear translocation. dFOXO is a transcription factor and its nuclear localization is key to its influences on growth, stress resistance, and metabolism [19]. The direct targets of dFOXO are conserved across several different mammalian tissues and species. Over-expression of nuclear localized dFOXO in fat body/gut extends longevity in flies and worms [20, 21]. In addition, overexpressing dFOXO in fly muscle extends lifespan [22].

Here, we investigate the effects and the mechanism of *rpd3* reduction on fly metabolism, stress resitance, and longevity. We found that flies with reduced *rpd3* levels have increased energy storage illustrated by increased levels of glucose, glycogen, trehalose, and triglycerides, which is consistent with their increased resistance to starvation. *rpd3* mutant flies have reduced IIS supported by decreased levels of *InR*, *chico*, and increased levels of *dfoxo* mRNA compared to controls. Genetic studies show an overlap between *rpd3* and IIS longevity pathways supported by a shorter life and reduced stress resistance of male flies with mutations in both *rpd3* and *dfoxo* compared to *rpd3* single mutant flies. Our data confer a novel link between *rpd3* and IIS and suggest IIS as a potential downstream mediator of the effects of *rpd3* mutation on fly health and metabolism.

## RESULTS

### *rpd3* reduction affects energy storage in flies

**Figure 1:**
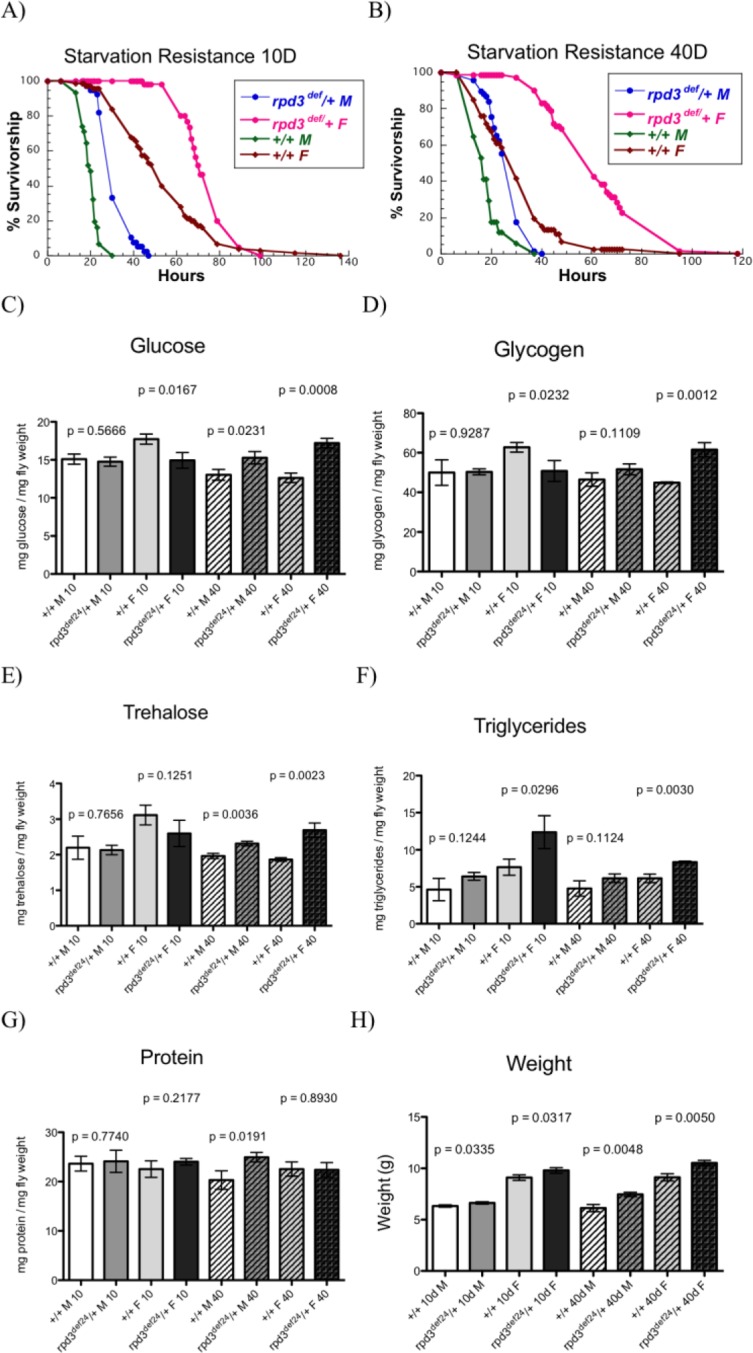
*rpd3* reduction affects stress resistance and metabolism. (A,B) Reduced *rpd3* levels increase stress resistance Survival curves for male and female *rpd3^def^/+* and control flies during starvation at age 10 (**A**) and 40 (**B**). (**C**-**G**) *rpd3* reduction affects intermediary metabolism: Total levels of glucose (**C**), glycogen (**D**), trehalose (**E**), triglyceride (**F**) and protein (**G**) in *rpd3^def^/+* and control male and female flies at age 10 and 40 days. (**H**) Weight of *rpd3^def^* and control flies used in C-G. Data are presented as means + SD (n=3, 30 flies per replicate. t test).

To examine the mechanism of the longevity extension observed in *rpd3* mutant flies we examined how Rpd3 reduction affects fly physiology by examining resistance to starvation, oxidative stress, and fly metabolism. We used two different heterozygous *rpd3* mutant alleles and their genetic controls, since homozygous *rpd3* mutation is embryonic lethal [23]. We used *rpd3* deficient (*rpd3^def^/+)* and their genetic controls, F1 progeny of *rpd3^def^/+* littermates. We also used *rpd3^P-UTR^/+* flies, an *rpd3* hypomorph, and *rpd3^P-1.8^/+*, which are genetic controls for *rpd3^P-UTR^/+* flies and have *rpd3* reduction only in the eyes [24]. Here we show that *rpd3^def^/+* mutant flies have higher starvation resistance at 10 and 40 days of age compared to control flies (Fig. 1 A,B; Supplemental Table 1A). Male *rpd3^def^/+* flies are 38% and 44% more resistant to starvation at ages 10 and 40 days, respectively. Female *rpd3^def^/+* flies are 28% and 108% more resistant at 10 and 40 days, respectively. To examine the potential mechanism of increased starvation resistance in *rpd3* alleles we examined the effects of *rpd3* reduction on fly metabolism. We quantified various forms of energy storage for the two *rpd3* mutant alleles. At 10 days of age *rpd3^def^/+* females have increased triglyceride levels, but reduced glucose and glycogen levels (Fig. 1C-G). At 40 days, *rpd3^def^/+* female flies have increased levels of glucose, glycogen, trehalose, and triglycerides. *rpd3^def^/+* males have increased levels of glucose and trehalose at 40 days, while no changes were observed at 10 days (Fig. 1C-F). Consistent with increased energy storage *rpd3^def^/+* flies weighed more than control flies (Fig. 1H).

### *rpd3^P-UTR^/+* females live longer during starvation due to metabolic adaptation

**Figure 2:**
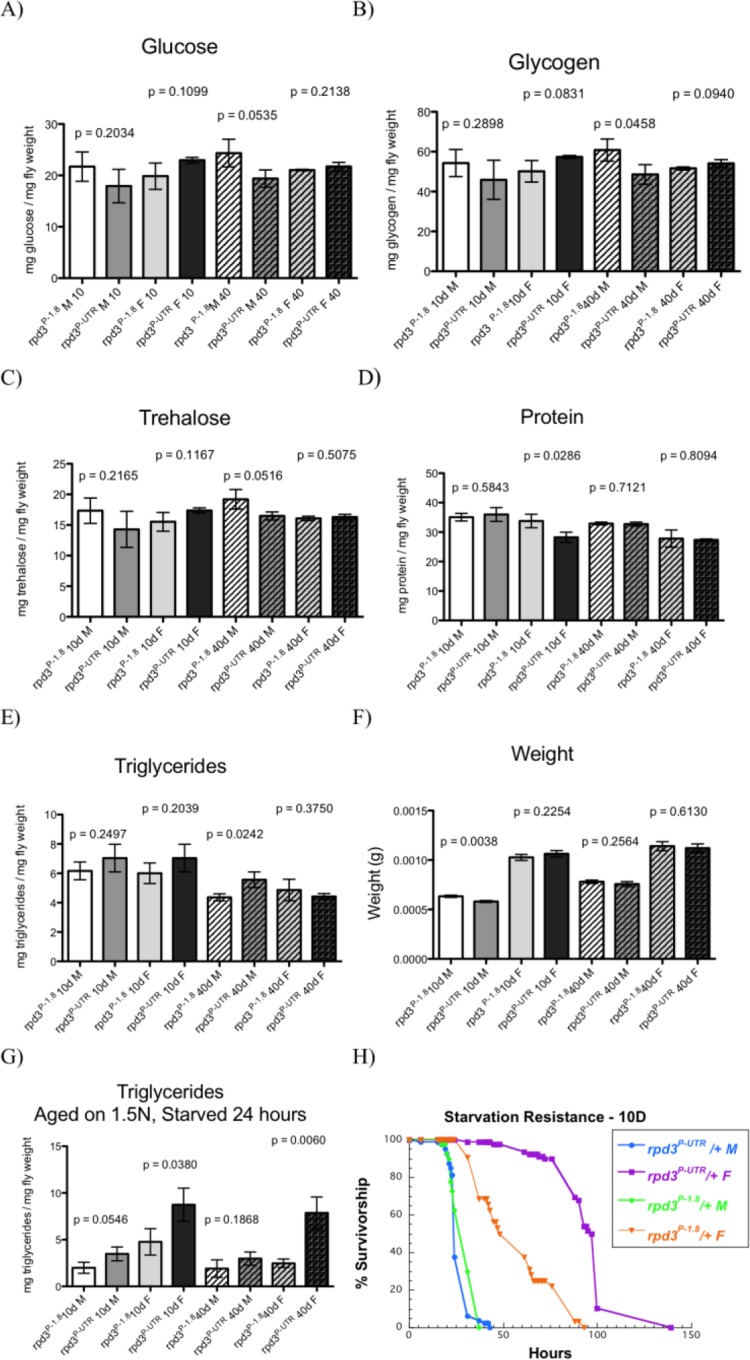
*rpd3* reduction affects fly intermediary metabolism (**A-E**) Total glucose (**A**), glycogen (**B**), trehalose (**C**), protein (**D**) and triglyceride (**E**) levels in *rpd3^P-UTR^/+* (experimental) and *rpd3^P-1.8^/+* (control) male and female flies at age 10 and 40 days. F) Weight of male and female flies used in (**C**-**E**). (**G)**. Total triglyceride levels in *rpd3^P-UTR^/+* and *rpd3^P-1.8^/+* flies aged on 1.5N for 10 or 40 days and then starved for 24 hours. Data are presented as means + SD (n=3, 30 flies per replicate. t test) (**H**) *rpd3* reduction increases stress resistance in flies. Survival curves of *rpd3^P-UTR^/+* and *rpd3^P-1.8^/+* male and female flies during starvation at 10 of age.

At age 10, male and female *rpd3^P-UTR^/+* flies have the same levels of energy stores as controls (Fig. 2A-F). At age 40, male *rpd3^P-UTR^/+* flies have increased levels of triglycerides but reduced trehalose, glucose, and glycogen. When kept on standard lab food, female *rpd3^P-UTR^/+* have the same triglyceride levels as controls at 40 days. To examine how *rpd3* reduction affects fly adaptation to starvation we kept flies at high calorie food (1.5N) for 10 or 40 days and then starved them for 24 hours. Triglyceride levels were much higher in *rpd3^P-UTR^/+* female, compared to levels found in *rpd3^P-1.8^/+* control female flies (Fig. 2G). Consistently, an increase in starvation resistance was observed in *rpd3^P-UTR^/+* flies when compared to *rpd3^P-1.8^/+* flies (Fig. 2H, Supplemental Table 1B). These data suggest that reduction in *rpd3* levels facilitates female adaptation to starvation, most likely by increasing efficiency in using metabolic reserves [25]. This is supported by our findings that *rpd3^P-UTR^/+* flies have increased starvation resistance and consistent with our recent report that *rpd3^P-UTR^/+* flies live longer compared to controls in conditions similar to starvation [11].

### *rpd3*-mutant flies have altered gene expression of components of the IIS pathway

**Figure 3:**
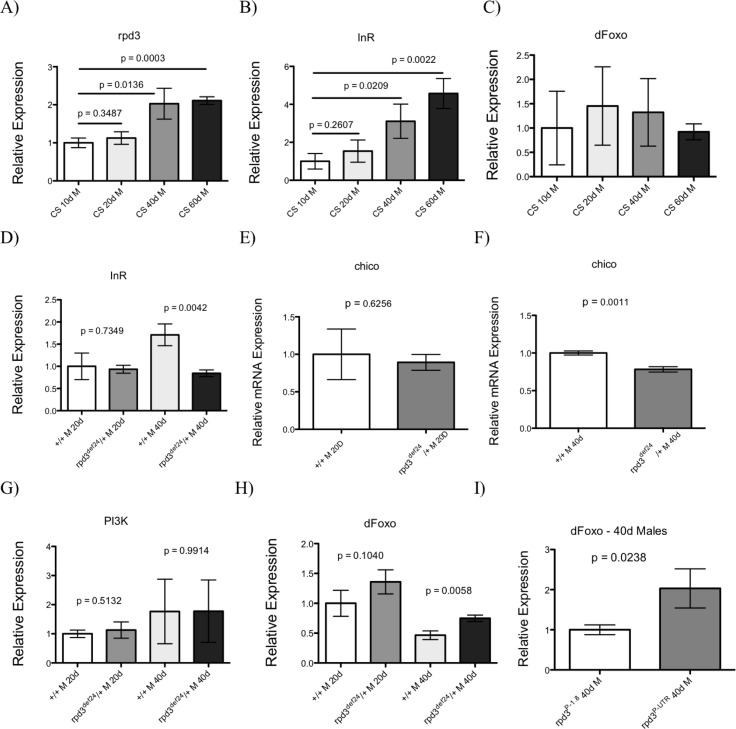
*rpd3* reduction decreases IIS. (A-C) Aging affects *rpd3* and *InR* mRNA levels The levels of *rpd3* (**A**), *InR* (**B**) or *dfoxo* (**C**) mRNA in the heads and thoraces of *Canton S (CS)* wild type male flies at 10, 20, 40 or 60 days determined by qPCR. Controls show an age-related increase in *rpd3* and *InR* mRNA levels. (**D-I**) The levels of *InR* (**D**), *chico* (**E**,**F**), PI3K (**G**), and *dfoxo* (**H**) mRNA in heads and thoraces of *rpd3^def^/yw* male flies at ages 20 and 40 and their genetic controls determined by qPCR. (**I**) The levels of *dfoxo* mRNA are increased in thoraces of *rpd3^P-UTR^/CS* male flies compared to *rpd3^P-1.8^/CS* controls at 40 days (n=3, **A**-**H**: 30 heads and thoraces per replicate. I: 30 thoraces per replicate. p as noted, t test).

The IIS pathway plays an important role in metabolism, stress resistance, and aging. Decreasing IIS by inhibiting *InR*, *chico*, or ablating the IPCs of the brain has been shown to extend the lifespan of fruit flies [26]. Overexpressing dFOXO, a downstream target of the IIS pathway, increases fly stress resistance. Since both reduction of *rpd3* and of different members of IIS pathway extend fly longevity and increase stress resistance, we examined if these two longevity pathways overlap. We first examined how aging affects transcriptional levels of *rpd3* and different members of IIS in a control strain of *Drosophila*, *Canton-S* (*CS*) at age 10, 20, 40, and 60 days. We found age-related increase in the levels of *rpd3* and *dInR* mRNA in the heads and thoraces of *CS* flies and no changes in mRNA levels of *dfoxo* (Fig. 3A-C). Next we examined the status of IIS in *rpd3* mutant flies and determined the levels of genes of members of the IIS pathway in the heads and thoraces (H + T) of *rpd3^def^/+* flies at 20 and 40 days of age. The mRNA levels of *InR* and *chico* were significantly decreased in heads and thoraces of *rpd3^def^* mutants compared to their genetic controls at age 40 (Fig. 3D-I). The mRNA levels of *PI3K*, another member of IIS pathway, were the same as in controls (Fig. 3G). dFOXO is a downstream effector of the IIS pathway, whose activity is inversely related to IIS. Consistently with reduced IIS in *rpd3* mutant flies, we found increased *dfoxo* mRNA expression in both *rpd3^def^/+* flies and *rpd3^P-UTR^/+* flies at 40 of age (Fig. 3H,I). We used only thoraces of *rpd3^P-UTR^* because their genetic controls *rpd3^P-1.8^* flies have reduced levels of *rpd3* mRNA in the eyes. Similarly, increased expression of *dfoxo* mRNA was found in whole body RNA isolated from heart-specific *rpd3* downregulation [12].

When the IIS is active, dFOXO is phosphorylated by Akt and localized in cytoplasm. Reduction in IIS decreases dFOXO phosphorylation, which allows nuclear dFOXO localization and activation of trans-cription of many genes known to mediate beneficial effects of reduced IIS. Our Western blots did not reveal any difference in the levels of phosphorylated dFOXO or in the ratio of nuclear and cytoplasmic fraction (Supplemental Fig. 1A-C). Nevertheless, this result could be due to low stability of the FOXO protein or possibly due to the quality of our anti-dFOXO antibody. Our data show that *rpd3* reduction prevents age-related increase in *InR* and reduction in *dfoxo* mRNA and suggest that decreased IIS could mediate the effects of *rpd3* mutation on *Drosophila* longevity.

### *dfoxo* partially mediates lifespan extension and increased stress resistance in *rpd3*-mutant flies

**Figure 4:**
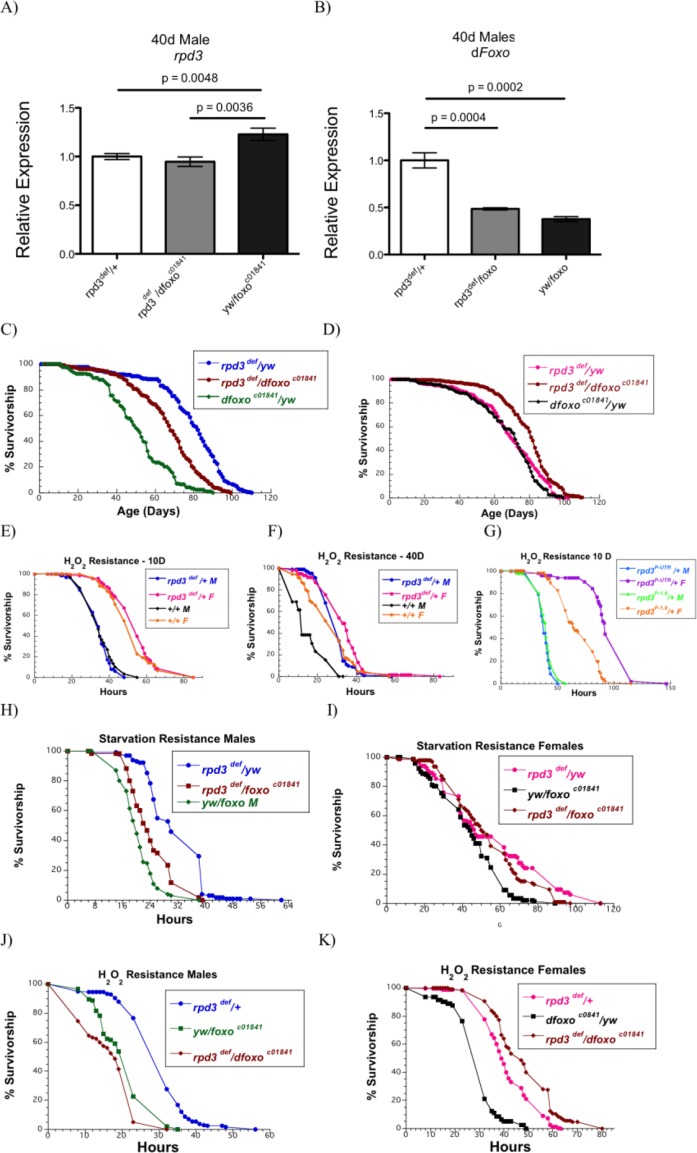
*dfoxo* partially mediates effects on longevity and stress resistance observed in male *rpd3* mutant flies (**A**, **B**) *rpd3* (**A**) and *dfoxo* (**B**) mRNA levels in the heads and thoraces of *rpd3^def^/yw, rpd3^def^/dfoxo^c01841^*and *dfoxo^c01841^/yw* male flies at 40 days determined by qPCR (n=3, 30 Heads and thoraces per replicate. p as noted, t test, error bars represent SEM). (**C**,**D**) Survival curves of male (**C**) and female (**D**) *rpd3^def^*/*yw*, *rpd3^def^/dfoxo^c01841^* and *dfoxo^c01841^/yw* flies. (**E-F)** Survival curves of *rpd3^def^/+* and control flies male and female flies on 5% H_2_O_2_ at age 10 (**E**) and 40 (**F**) days. (**G**) Survival curves of *rpd3^P-UTR^/+* and *rpd3^P-1.8^/+* male and female flies exposed to 5% H_2_O_2_ at age 10 days of age. (**H**, **K**) Survival curves for male (**H**, **J**) and female (**I**, **K**) *rpd3^def^*/*yw*, *rpd3^def^/dfoxo^c01841^* and *dfoxo^c01841^/yw* flies during starvation (**H**, **I**) or on 5% H_2_O_2_ (**J**, **K**) at age 40.

To strenghten the link between *rpd3* mutation and reduced IIS, we generated flies that were double mutants for both *rpd3* and *dfoxo* and examined their longevity and stress resistance. *dfoxo* is the downstream IIS target that mediates beneficial effects of reduced IIS signaling. We confirmed that the levels of *rpd3* and *dfoxo* expression were reduced based on the presence of the mutations in male flies (Fig. 4A,B). *rpd3^def^*/*yw* mutants and *rpd3^def^/dfoxo^c01841^* double mutants had lower levels of *rpd3* mRNA expression compared to *dfoxo^c01841^/yw* mutant flies (Fig. 4A). *dfoxo^c01841^/yw* and *rpd3^def^/dfoxo^c01841^* males have decreased levels of *dfoxo* mRNA expression compared to *rpd3^def^*/*yw* flies (Fig. 4B). Notably, male *rpd3^def^*/*dfoxo^c01841^* double mutant flies have higher levels of *dfoxo* mRNA compared to single *dfoxo^c01841^/yw* flies, consistent with our data that *rpd3* mutation increases *dfoxo* mRNA levels (Fig. 4B). Lifespan experiments were performed on these flies. Heterozygous *rpd3^def^/+* males lived the longest (Fig. 4C, Supplemental Table 2) *dfoxo^c01841^*/*yw* males had the shortest lifespan, whereas *rpd3^def^/dfoxo^c01841^* double mutants had a longevity in the middle of these two. These data suggest that the full beneficial effects of *rpd3* mutation on fly longevity require both copies of *dfoxo*, and that small increases in *dfoxo* levels in *rpd3^def^*/*dfoxo^c01841^*may increase longevity of double mutant flies compared to *dfoxo^c01841^/yw* males. In contrast, female *rpd3^def^*/*dfoxo^c01841^* flies live longer compared to single mutant flies, which have similar lifespans. The differences between male and female in longevity effects of double mutations may be due to sexual dimorphism previously described to be associated with IIS, FOXO, and p53 [27]. Similar differences were observed in stress response, which is shown in next paragraph and discussed later.

### Increased stress resistance in flies with reduced *rpd3* and IIS

Reduced IIS is associated with increased resistance to oxidative stress. Since *rpd3* reduction is associated with IIS reduction we examined if flies with reduced *rpd3* levels are more resistant to hydrogen peroxide (H_2_O_2_). Male and female *rpd3^def^/+* are more resistant to H_2_O_2_ compared to control flies at 40 days of age, while no difference was observed at age 10 days (Fig. 4E,F; Supplemental Table 3). Female *rpd3^P-UTR^/+* flies are more resistant to H_2_O_2_ at both ages but no difference was observed in male *rpd3^P-UTR^/+* flies (Fig 4G, Supplemental Table 3 and data not shown). To examine the role of *dfoxo* in increased stress resistance of *rpd3* mutant flies, we determined starvation and H_2_O_2_ resistance of *rpd3* mutant flies with or without *dfoxo* mutation. Similarly to longevity studies, *rpd3^def^/+* male flies had the highest mean survival rate when exposed to starvation at 40d of age (Fig. 4H,I; Supplemental Table 4). *rpd3^def^/dfoxo^c01841^* double mutants had a lower mean survival, but they were more resistant to starvation than *dfoxo^c01841^/yw* flies (Fig. 4H,J). Similarly, male *rpd3^def^/+* mutants are more resistant to H_2_O_2_ compared to *rpd3^def^/dfoxo^c01841^* or *dfoxo^c01841^*/*yw* flies. These results suggest that *dfoxo* mediates some of the effects of *rpd3* mutation on lifespan in *Drosophila* males and is required for full beneficial effects of *rpd3* mutation on longevity and stress resistance. Female flies double mutant for *rpd3^def^* and *dfoxo^c01841^* live longer and have similar starvation and H_2_O_2_ resistance compared to single *rpd3^def^/+*, but are more resistance compared to *dfoxo^c01841^/yw* mutant flies (Fig. 4D,I,K; Supplemental Table 5).

## DISCUSSION

### *rpd3* reduction affects fly metabolism and IIS

Members of the class I HDAC family are vital regulators of chromatin structure and gene expression. They have multiple functions including a role in development, metabolism, and aging [1, 28]. Deletion of *rpd3* in yeast extends their replicative lifespan [7]. Flies heterozygous for a null or a hypomorphic *rpd3* mutant alleles had an extended lifespan compared to genetic controls [9, 11]. Moreover, heart-specific *rpd3* downregulation in flies increases heart function, stress resistance, and extends longevity [12]. In addition, HDAC inhibitors trichostatin A and butyrate also extend fly lifespan [13, 29]. Longevity extension of *rpd3* mutant flies was not further extended by dietary restriction (DR) and was absent in flies double mutant for *dSir2* and *rpd3* mutations [30]. Both DR flies and flies with reduced *rpd3* have increased *dSir2* levels [12, 30]. These findings suggested that the mechanism of longevity extension in *rpd3* mutant and DR flies overlap. However, the full understanding of the mechanism of longevity extension associated with *rpd3* reduction is missing. Therefore we examined intermediary metabolism and starvation resistance of two *rpd3* heterozygous mutant alleles. Increased starvation resistance in female *rpd3^def^/+* flies is the result of increased energy storage in forms of triglyceride at young and old age, as well as increased carbohydrate levels at age 40. Metabolic adaptation to fasting is key to preserving homeostasis of an organism. This adaptation includes mobilization of lipids followed by their oxidation into ketone bodies, which are used as a source of energy in other tissues. Metabolic adaptation to starvation in *rpd3* mutant flies is illustrated by the findings that *rpd3* reduction prevents a fasting-induced decrease in triglycerides in female *rpd3^P-UTR^/+* flies. This is consistent with our recent report that *rpd3^P-UTR^/+* flies live longer compared to controls in conditions similar to starvation and findings that heart-specific Rpd3 downregulation increases fly resistance to starvation at age 2 days [11, 12]. To get insights into the mechanism associated with *rpd3* reduction we examined if these changes are mediated by IIS. The IIS pathway is a nutrient sensing pathway, which also affects the activity of metabolic enzymes. When nutrients are abundant IIS is active, dFOXO is phosphorylated, and it is localized in the cytoplasm. Reduced IIS decreases phosphorylation of dFOXO, which promotes dFOXO nuclear translocation. In the nucleus, dFOXO regulates glucose, glycogen, and lipid metabolism by activating transcription of key enzymes involved in these metabolic pathways. For instance, dFOXO activates glycogenolysis and gluconeogenesis by activating transcription of glucose-6-phosphatase (G6P) and phosphoenolpyruvate carboxykinase (PEPCK) mRNA, respectively [31]. dFOXO regulates autophagy in response to starvation, which promotes recycling of the cellular components [32]. Intriguingly, adult dFOXO null mutant flies have no difference in starvation resistance or energy storage [33]. Under conditions when nutrients are limited, activated dFOXO upregulates *InR* transcription. This allows the cells to accumulate *InR* mRNA, and prime them to respond quickly when nutrients become available [34]. Once IIS is activated, it will upregulate growth and inhibit dFOXO activity via its phosphorylation. Here we show that in a control strain of *Drosophila*, *CS*, the levels of *InR* mRNA expression gradually increase throughout the lifespan. We found that *rpd3* reduction affects the IIS pathway and prevents age-associated changes in the transcript levels of IIS genes. *rpd3^def^* flies have reduced levels of *InR* and *chico* and increased *dfoxo* mRNA at 40 days. These changes in IIS are consistent with metabolic changes found in 40 day old *rpd3* mutant flies.

### dFOXO mediates some longevity effects observed in *rpd3* mutant flies

Our genetic studies suggest that dFOXO mediates some longevity effects of *rpd3^def^* mutant flies. This is illustrated by shorter lifespan of male flies double mutant for *rpd3* and *dfoxo* compared to *rpd3^def^/+* flies. *rpd3^def^*/*dfoxo^c0184^* flies live longer compared to *dfoxo* single mutant flies, which is most likely mediated by higher *dfoxo* mRNA expression in double mutants compared to *dfoxo* single mutants. Similarly, *rpd3^def^*/*dfoxo^c0184^* flies have reduced resistance to H_2_O_2_ compared *rpd3^def^/+* indicating that increased dFOXO mediates resistance to stress in *rpd3* mutant male flies. This is consistent with findings that overexpression of nuclear localized dFOXO mediates increased resistance to oxidative stress in flies and mammalian cells [33].

Moreover, treatment with PBA, a HDAC1 inhibitor, increases expression of genes that have been implicated in response to oxidative stress, such as SOD, gluthathione S-transferase, and heat-shock protein. However, female *rpd3^def^/dfoxo^c01841^* live longer and are similarly more resistant to H_2_O_2_ compared to both single *rpd3^def^/+* and *dfoxo^c01841^/+* mutant flies. It is possible that reduced *rpd3* levels in *rpd3^def^/+* female flies increase *dfoxo* levels of the remaining wild type copy of the gene, which contribute to longer lifespan. The differences may be also due to sexual dimorphism previously described to be associated with IIS, FOXO, and p53. It was found that nervous-system specific overexpression of *p53* increases female lifespan in a *foxo* null background. In contrast, in males *foxo* null mutation caused the tissue-specific effects of p53 [27]. Several studies have examined the relationship between IIS and HDAC inhibition. β–hydroxybutyrate (βOHB) is an endogeneous inhibitor of HDACs 1, 3, and 4 (Class I and Class II). βOHB is one of the ketone bodies released during fasting and exercise. βOHB inhibition of HDAC1 and HDAC2 activity results in increased histone acetylation and gene expression. Particularly important is induction of Foxo3, the mammalian orthologue of the dFOXO, expression by removing HDAC-mediated *Foxo3* repression via hypoacetylation of its promoter [35]. Ye reviewed the effects of an HDAC1 inhibitor on energy metabolism and insulin sensitivity [36]. It was also reported that use of butyrate, a class I and II HDAC inhibitor, improves glucose metabolism and prevents age-related atrophy [37]. However, less is known about the specific role of *rpd3* reduction on metabolism in flies. The data presented here provide new information about the effects of *rpd3* reduction on fly metabolism and link the changes in metabolism to a reduction in IIS. Taken together, our genetic data strenghten the link between *rpd3* reduction and reduced IIS.

**Figure 5:**
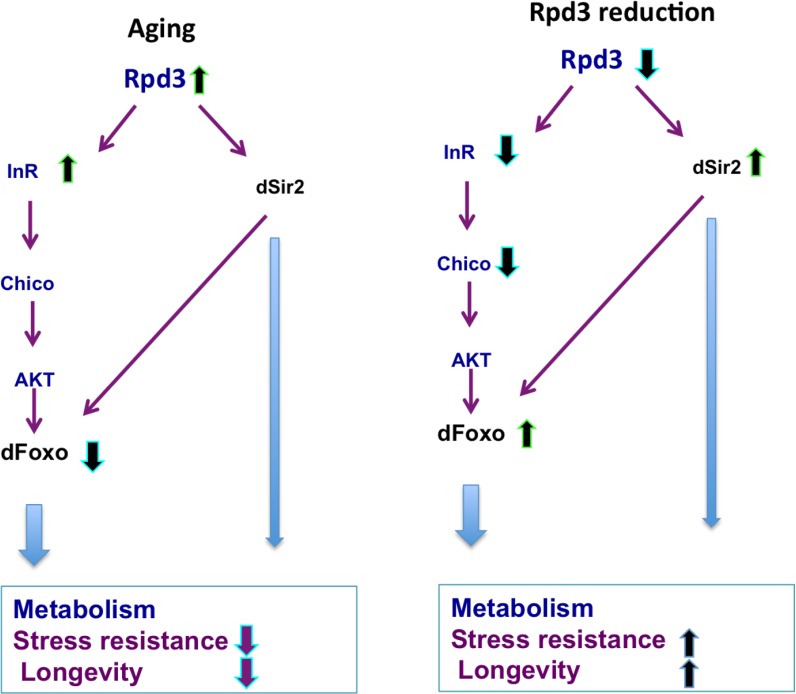
*rpd3* reduction prevents age-associated changes in IIS Age-associated increases in the *rpd3*, *InR,* and a decrease in *dfoxo* mRNA are observed in wild type flies, Reduced *rpd3* activity decreases *InR* and *chico* mRNA, while increases *dfoxo* and dSir2 mRNA levels. Reduced *rpd3* affects metabolism, increases stress resistance and longevity by reducing IIS and increasing *dSir2* levels. Purple and light blue arrows indicate downstream effects, green arrows represent increase and blue reduction in mRNA levels.

Longevity extension in *rpd3* mutant flies has been linked to dSir2, 4E-BP, dFOXO, and CR longevity pathways [9, 11, 12, 30]. The data presented here add to our understanding of the mechanism of *rpd3*'s effects on longevity by identifying a novel genetic link between *rpd3* reduction and IIS (Fig. 5). However, considering that *rpd3* has multiple targets, the reduction of *rpd3* does not completely reproduce phenotypes of the flies with reduced IIS. In *Drosophila*, reduction in IIS results in female sterility and longevity extension independent of fertility [15,16]. *Chico* mutants are smaller and sterile, but they contain twice as much lipid content per weight as do genetic controls [38]. Overexpression of *dfoxo* in adult fat body and the gut has no effect on fecundity, total triglyceride, total trehalose, or total glycogen content, but it reduced fly weight and total protein content [39]. *rpd3* mutant flies have reduced levels of *InR* and *chico*, increased levels of *dfoxo* mRNA, and increased resistance to starvation and oxidative stress. However, *rpd3* flies are as fertile as controls on a standard diet, and their weight is higher or the same compared to controls. These differences highlight the complex mechanism of the beneficial effects of *rpd3* reduction on fly health and longevity.

Here we show that *rpd3* reduction affects fly metabolism characterized by increased energy storages, higher resistance to starvation and oxidative stress, and increased longevity. These effects are mediated at least partially by reduced IIS, confirmed by genetic studies showing that longevity extension requires increased dFOXO (Fig. 5). Previous studies highlight the role of dSir2 in *rpd3* effects on lifespan [12, 30]. Histone modifying enzymes link changes in nutrient availability to changes in intermediary metabolism by affecting the activity and stability of the enzymes involved in glycogenesis, glycolysis, gluconeogenesis, and β-oxidation through acetylation [40–42]. The acetylation pattern differs in tissues and cell types, suggesting a complex, highly orchestrated regulation of acetylation levels at the organismal level. Future studies on the acetylation status of enzymes involved in intermediary metabolism in different tissues of *rpd3* mutant flies would further expand our knowledge of the effects of *rpd3* reduction on metabolism, health, and longevity. Our data illustrates how these complex interactions result in phenotypic changes at the organismal level. Further studies are necessary to examine how tissue-specific alterations in *rpd3* levels orchestrate these changes.

## MATERIALS AND METHODS

### Fly strains and maintenance

*rpd3-deficient (rpd3^def24^*) and *rpd3*–hypomorphic (*rpd3^P-UTR^*) flies and their genetic controls were used in the experiments*.* Genetic controls for *rpd3^def24^* were progeny generated by crossing F1 *rpd3^def24^/+* littermates. The hypomorphic *rpd3^P-UTR^* allele has a P-element inserted in the 5′UTR region of the *rpd3* genes, which affects expression throughout the fly's bod. The control *rpd3^P-1.8^* allele has a P-element inserted 1.8 Kb upstream from the transcriptional start site, which only decreases expression in the eye [24]. *Canton S*, *yw*, and *dfoxo^c01841^* were kindly provided by the Bloomington Stock center. *dfoxo^c01841^* flies were backcrossed to *yw* strain for 10 generations to eliminate difference in genetic background. Flies were collected within 24 hours of eclosion and maintained using standard culture media in plastic vials. They were kept at 25°C in a humidified incubator. About 25 males and 25 females were kept together in each vial, and they were passed to a fresh vial every Monday, Wednesday, and Friday. Standard corn and 1.5N (High calorie diet) diet was prepared as previously reported [43].

### Lifespan studies

Flies were collected within 24 hours of eclosion and maintained as described above. 25 males or 25 females were placed in each vial. They were passed every 2 days up to age 30 days and every day after that and the number of dead flies were counted. The number of flies in each survivorship study is listed in Tables 1-7.

### Starvation and oxidative stress resistance

Flies were collected as described above and aged until 10 or 40 days of age. They were separated into vials of 20 males or 20 females and transferred into new vials containing 2 filter papers with 300 μl of DI H_2_O for starvation studies. 300 μl of 5% H_2_O_2_ with 5% sucrose was used instead of water for oxidative stress studies following initial 6 hours of starvation. The number of dead flies was counted at regular intervals, and the vials were changed ever 24 hours.

Resistance to paraquat: flies were collected as described above. 20 flies were transferred to a vial containing filter paper soaked with 300 μL of 20 mM paraquat following initial starvation for 6 hours. The number of dead flies was counted hourly during the day and twice overnight until no flies remained alive. Stress resistance data were analyzed by log-rank tests using the JMP 10 program. Total number of flies per experiment is listed in Supplemental Tables 1, 2, and 3.

### Biochemistry

Flies were collected and aged as described above. At ages 10 and 40 days, flies were weighed, homogenized, and 25 μl of homogenate was aliquoted into 96-well plates. For glucose, PGO enzyme plus color reagent was added to each well, the plate was incubated at 37°C, and the optic density was read at 450 nm. For glycogen, the procedure was the same except amyloglucosidase was added to each well in addition to the other enzyme. For trehalose, the procedure was the same as glucose except the samples were incubated with trehalase before addition of PGO. For protein, BioRad protein solution was diluted 1:5, added to each well, and then the plate was read at 595 nm. For triglycerides, free glycerol and triglyceride reagents were added to each well, the plate was incubated at 37°C, and the optic density was read at 540 nm [45, 46].

### Quantitative PCR (qPCR)

Flies were frozen at the appropriate age, and RNA was isolated from the heads and thoraces, thoraces, or heads. cDNA was synthesized from RNA. Using TaqMan primers and the Applied Biosystems Thermal Cycler, levels of gene expression were determined. The data were normalized to the results of the control flies using *ankryn* as standard [47].

### Western blots

40 day old flies were dissected on CO_2_ and placed in tubes containing Kinase Lysis Buffer. The tissue was homogenized using Teflon pestles. Protein concentrations were quantified using BioRad Protein determination Kit. Lysates were cleared by centrifugation, run on a Lithium Dodecyl Sulfate (LDS) PAGE using NuPAGE NOVEX 4-12% gradient gels (Life Technologies), and transferred overnight onto nitrocellulose membranes in transfer buffer (20mM CAPS pH 11, 20% MeOH). Western blotting was performed using standard procedures with washes done in TBST. Membranes were blocked in 5% milk for an hour followed by overnight, 1:500 primary antibody incubation at 4°C. Secondary antibody incubation was done for an hour at room temperature at a concentration of 1:5,000. Secondary antibodies were labeled with Horseradish Peroxidase. Blots were imaged using the Kodak Image Station 4000 MM following application of Enhanced Chemiluminensence reagent (Perkin Elmer). The dFOXO antibody was a gift from Mark Tatar, and the two anti-phospho-Foxo3A antibodies (Cat #9466) were gifts from Cell Signaling Technology to test if they would work in *Drosophila* tissue. Nuclear and cytoplasmic fractions were isolated from 40-day old flies following instruction for The Active Motif Nuclear Extract Kit (Carlsbad, CA. USA, Cat #40010).

### Statistical analysis

Significance was determined using a two-tailed, unpaired t-test from at least three independent experiments and expressed as P values. P < 0.05 is considered to be significant. P values are specifically indicated in each figure. Error bars represent standard deviation (SD). Longevity data were censored for early mortality (1-9 Days) and analyzed by log-rank tests using the JMP 12 program.

## SUPPLEMENTARY MATERIAL FIGURE AND TABLES


