# Supplementary material for: Rpd3 interacts with insulin signaling in Drosophila longevity extension
Source: Aging (Albany NY). 2016 Nov 14;8(11):3028–40. doi: 10.18632/aging.101110 (PMC5191884; doi:10.18632/aging.101110)
Supplement: Supplementary file 1 [file aging-08-3028-s001.pdf]

SUPPLEMENTAL MATERIAL

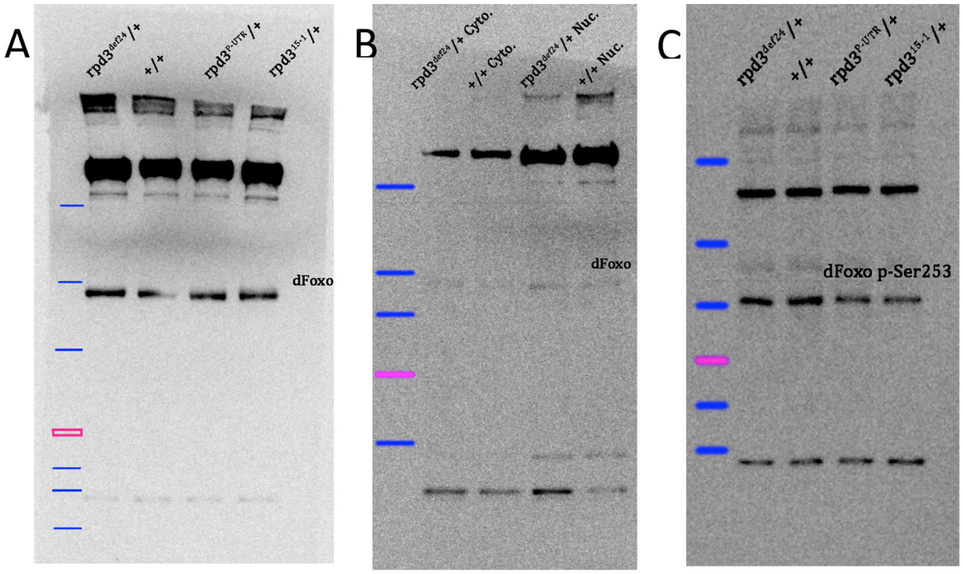

Supplemental Figure 1. (**A**) Western blot staining for dFOXO in 40 day old *rpd3*^{def24}*/+*, control (*+/+)*, *rpd3*$^{P\text{-}UTR}$*/CS* and *rpd3*$^{P\text{-}1.8}$*/CS* male flies. (**B**) Western blot staining for dFOXO in the cytoplasmic and nuclear fractions of 40 day old *rpd3*^{def24}*/+* and control (*+/+)* male flies. (**C**) Western blot staining for phosphorylated Serine 253 dFOXO in 40 day old *rpd3*^{def24}*/+*, control (*+/+*), *rpd3*$^{P\text{-}UTR}$*/CS*, and *rpd3*$^{P\text{-}1.8}$*/CS* (control) male flies.

Supplemental Table 1A. Starvation resistance of *rpd3*^{def}*/+* heterozygous flies and genetic controls.

Gender	Genotype	N	Age	Mean LS (% change)	Median LS	X^2	p	Maximal life span (% change)
M	*rpd3*^{def}*/+*	39	10	32 (38)	30	54.82	<0.0001*	44 (40)
M	*Control*	31	10	20	20			26
F	*rpd3*^{def}*/+*	60	10	74 (28)	53	24.880	<0.0001*	92 (2)
F	*Controls*	75	10	54	72			90
M	*rpd3*^{def}*/+*	69	40	27 (44)	30	20.9381	<0.0001*	37 (11)
M	*Control*	17	40	18	17			34
F	*rpd3*^{def}*/+*	71	40	64 (108)	61	70.1045	<0.0001*	96 (54)
F	*Controls*	46	40	31	30			63

The mean, median, and maximal lifespan of *rpd3*^{def}*/+* and genetic control (+/+) heterozygous male (M) and female (F) flies after exposure to starvation at 10 or 40 days of age. Control values are compared to either male or female *rpd3*^{def}*/+* groups to determine the percent change in mean, median, and maximal lifespan. Mean, median, and maximal lifespan are in hours. Log-rank analyses were performed using the JMP 12 program. M = Males, F = Females, N = number of flies in the experiment.

Supplemental Table 1B. Starvation resistance of *rpd3^{P-UTR}* heterozygous flies and genetic controls *rpd3$^{P-1.8}$/+* flies.

Gender	Genotype	N	Age	Mean LS (% change)	Median LS	X^2	p	Maximal life span (% change)
M	*rpd3^{P-UTR}/+*	80	10	32 (9)	31	3.6315	0.0567	38
M	*rpd3$^{P-1.8}$/+*	40	10	29	31			37
F	*rpd3^{P-UTR}/+*	78	10	96 (40)	99	81.6546	<0.0001*	139 (36)
F	*rpd3$^{P-1.8}$/+*	32	10	57	55			89

The mean, median, and maximal lifespan of *rpd3^{P-UTR}/+* and their genetic control *(rpd3$^{P-1.8}$/+)* heterozygous male (M) and female (F) flies after exposure to starvation at 10 days of age. Control values are compared to either male or female *rpd3^{P-UTR}*/+ to determine the percent change in mean and maximal lifespan. Mean and median lifespan are in hours. N = number of flies in the experiment. Log-rank analyses were performed using the JMP 12 program.

Supplemental Table 2. *dfoxo* is required for longevity extension in *rpd3^{def}* mutant flies

Gender span	Genotype	N	Mean (% change)	Median LS	X^2	p	Maximal life (% change)
M	*rpd3^{def}/+*	180	77.7	82			101.1
M	*rpd3^{def}/dfoxo ^{c01841}*	225	65.6 (-16)	68	65.99	<0.0001*	91.5 (-9.5)
M	*dfoxo ^{c0184}/ +*	123	50 (36)	51	176.40	<0.0001*	76.7 (-24)
F	*rpd3^{def}/+*	200	68.6	70.5			93.7
F	*rpd3^{def}/dfoxo ^{c0184}*	238	78.8 (-15)	82	27.868	<0.0001*	101.2 (8)
F	*dfoxo ^{c0184}/ +*	132	67 (2)	72	2.474	0.1158	91.4 (3)

The mean, median, and maximal lifespans of *rpd3^{def}/+, rpd3^{def}/dfoxo^{c01841}*, and *dfoxo^{x01841}/+* heterozygous male (M) and female (F) flies. *dfoxo^{x01841}* flies are hypomorphic for *dfoxo.* All values are compared to either male or female *rpd3^{def}/+* groups to determine the percent change in median and maximal lifespan. Data are censored for 0-10 days. N: number of flies used in the experiment. Median and maximal lifespan are in days. Log-rank analyses were performed using the JMP 12 program.

Supplemental Table 3. Heterozygous flies with reduced *rpd3* mRNA levels have increased resistance to H_2O_2 compared to the genetic control flies at 40 days.

Gender	Genotype	N	Age	Mean (% change)	Median LS	X^2	p	Maximal life span (% change)
M	$rpd3^{def}/+$	65	10	34 (-2.5)	34	0.4934	0.4824	43 (-8.5)
M	*Control*	30	10	35	34			49
F	$rpd3^{def}/+$	73	10	55 (4.9) 55	1.3942	0.2377		80 (2.7)
F	*Controls*	79	10	52	55			78
M	$rpd3^{def}/+$	53	40	25 (155.8)	24	31.3716	0.0001*	31 (17.8)
M	*Control*	31	40	15	12			26
F	$rpd3^{def}/+$	55	40	39 (39.5)	36	5,7003	0.0170*	56 (-7.9)
F	*Controls*	39	40	28	31			60
M	$rpd3^{P\text{-}UTR}/+$	58	10	39 (-3)	38	1.4847	0.2230	48 (-60
M	$rpd3^{P\text{-}1.8}/+$	56	10	40	40			51
F	$rpd3^{P\text{-}UTR}/+$	52	10	97 (30)	92	55.2690	<0.0001*	116 (20)
F	$rpd3^{P\text{-}1.8}/+$	57	10	68	64			94

The mean, median, and maximal lifespan of *rpd3*^{def}/+ and genetic control (+/+), *rpd3* $^{P\text{-}UTR}$ /+, and genetic control (*rpd3* $^{P\text{-}1.8}$/+) heterozygous male (M) and female (F) flies after exposure to H_2O_2 at 10 or 40 days of age. Control values are compared to either male or female *rpd3*^{def}/+ groups to determine the percent change in mean and maximal lifespan. Mean and median lifespan are in hours. N = number of flies in the experiment. Log-rank analyses were performed using the JMP 12 program.

Supplemental Table 4. *dfoxo* is required for increased starvation resistance in *rpd3*^{def} heterozygous flies at 40 days.

Gender	Genotype	N	Mean (% change)	Median LS	X^2	p
M	$rpd3^{def}/yw$	102	29	29		
M	$dfoxo^{c01841}/yw$	101	20 (-31)	20	82.3659	<0.0001
M	$rpd3^{def}/yw$	102	29	29		
M	$rpd3^{def}/dfoxo^{c01841}$	60	24 (-17)	23	16.1367	<0.0001
F	$rpd3^{def}/yw$	83	55	47		
F	$dfoxo^{c01841}/yw$	94	53 (-4)	51	1.7	0.18
F	$rpd3^{def}/yw$	83	55	47		
F	$rpd3^{def}/dfoxo^{c01841}$	90	44 (20)	45	16.3721	<0.0001*

The mean and median lifespans of *rpd3*^{def}/+, *rpd3*^{def}/*dfoxo*^{c01841}, and *dfoxo*^{c01841}/+ heterozygous male (M) and female (F) flies. *dfoxo*^{c01841} flies are hypomorphic for *dfoxo.* All values are compared to either male or female *rpd3*^{def}/+ groups to determine the percent change in mean and median lifespan. Mean and median lifespan are in hours. The similarity in stress resistance between *rpd3*^{def}/*dfoxo*^{c01841} and *dfoxo*^{c01841}/+ male and female flies was also determined. N: number of flies used in the experiment. Three independent experiments with similar results were combined. Mean and Median lifespans are in hours. Log-rank analyses were performed using the JMP 12 program.

Supplemental Table 5. *dfoxo* is required for increased H_2O_2 resistance in *rpd3*^{def} heterozygous flies.

Gender	Genotype	N	Mean (% change)	Median LS	X^2	p	Maximal life span (% change)
M	*rpd3*^{def}/+	166	30	32			42
M	*rpd3*^{def}/*dfoxo*^{c01841}	82	17 (43)	18	139.6062	<0.0001*	24 (42)
M	*dfoxo*^{c01841}/+	88	21 (30)	23	82.7128	<0.0001*	32 (22)
F	*rpd3*^{def}/+	166	41	40			57
F	*rpd3*^{def}/*dfoxo*^{c01841}	128	48 (-17)	47	26.7491	<0.0001*	71 (-20)
F	*dfoxo*^{c01841}/+	80	30 (27)	32	89.8302	<0.0001*	43 (25)

The mean and median lifespans of *rpd3*^{def}*/+, rpd3*^{def}*/dfoxo*^{c01841}, and *dfoxo*^{c01841}*/+* heterozygous male (M) and female (F) flies after exposure to H_2O_2 at 40 days of age. *Dfoxo*^{c01841} flies are hypomorphic for *dfoxo.* All values are compared to either male or female *rpd3*^{def}*/+* groups to determine the percent change in mean or median lifespan. N: number of flies used in the experiment. Three independent experiments with similar results were combined. Mean and median lifespan are in hours. Log-rank analyses were performed using the JMP 12 program.
